# Self-Reported Reasons for Activity Limitations According to Age and Sex in Community-Dwelling Stroke Survivors

**DOI:** 10.3390/healthcare11101420

**Published:** 2023-05-14

**Authors:** Young-Ah Choi, Yeo Hyung Kim

**Affiliations:** 1 Department of Rehabilitation Medicine, Incheon St. Mary’s Hospital, College of Medicine, The Catholic University of Korea, Seoul 06591, Republic of Korea; simple16@catholic.ac.kr; 2 Department of Rehabilitation Medicine, College of Medicine, The Catholic University of Korea, Seoul 06591, Republic of Korea

**Keywords:** age, cerebrovascular disorders, activity limitation, sex, stroke

## Abstract

We examined self-reported reasons for activity limitations among Korean community-dwelling stroke survivors, focusing on age and sex differences. Data from 1547 stroke survivors who participated in the Korean National Health and Nutrition Examination Survey were analysed. The study outcomes were the self-reported reasons for activity limitations, encompassing general medical factors and stroke-related problems. These reasons were compared by age (<65 vs. ≥65 years) and sex using a complex-sample chi-square test. Stroke survivors reported different musculoskeletal, medical, and neurological problems as reasons for activity limitations, which differed by age and sex. Older stroke survivors reported more problems related to dementia, memory loss, auditory problems, back or neck problems, arthritis, or leg pain than younger survivors. Women reported more psychiatric problems, headaches or dizziness, back or neck problems, arthritis, gastrointestinal problems, and dental or oral problems than men. Older and female stroke survivors reported a higher mean number of reasons for activity limitations compared to younger and male survivors. Thus, a tailored approach considering age and sex is necessary to help stroke survivors with activity limitations in the Korean community. This study highlights the importance of considering demographic factors when designing interventions to improve their quality of life.

## 1. Introduction

Stroke is a leading cause of long-term disability worldwide and can have marked adverse effects on physical and mental function [1]. The International Classification of Functioning, Disability, and Health (ICF) is a conceptual model which integrates medical and social components that influence the impact of strokes on patients. A stroke can impair the functions and structures of the body, which may result in challenges in performing everyday activities, and consequently limit the range of activities that the patient can perform [2]. Activity limitation represents a human characteristic that reflects medical, functional, and social perspectives. Due to the recent advances in acute stroke treatment, the increasing number of stroke survivors has led to a rise in the burden of post-stroke activity limitations, disabilities, and participation restrictions, making it a critical public health issue [3]. Previous studies that monitored cohorts of stroke survivors have reported that a substantial proportion of subjects, ranging from 36% to 57.6%, continued to experience activity limitations for several years after a stroke [4]. Furthermore, activity limitations are significantly more prevalent among individuals who have experienced a stroke compared with individuals without any chronic health conditions, as well as individuals with non-stroke-related chronic conditions [5,6].

Environmental, psychosocial, sociodemographic, and medical factors are known to have significant effects on activity limitations in the general population [7,8]. Survey research examining the association of limitations in activities of daily living (ADL) with various self-reported health conditions among older Singaporeans showed that joint/nerve pain, stroke, pelvic/femoral fractures, heart diseases, diabetes, osteoporosis, chronic respiratory illness, and renal/urinary tract illness revealed a significant association with ADL limitations [9]. Moreover, several factors, including age, stroke severity, comorbidities, emotions, and extremity function, have been linked to post-stroke impairments and activity limitations [10,11]. Therefore, identification of the factors associated with activity limitations among stroke survivors can be helpful in developing interventions.

Prior studies have suggested that biological factors, such as age and sex, play a role in the prognosis of post-stroke outcomes and may be closely related to activity limitations after stroke [12,13]. A cohort study found that older age was the main contributor to activity limitations, and women were functionally more disabled than men [14]. A French study revealed a higher incidence and greater severity of limitations in ADL and instrumental ADL with increasing age among self-reported stroke participants [5]. Moreover, a previous systematic review suggested that women tended to experience more pronounced long-term limitations in activity compared with men [15]. However, to our knowledge, few studies have investigated the self-reported reasons for activity limitations from the stroke survivors’ perspectives, despite the relatively subjective nature of such studies, compared to impairment.

Among the factors potentially associated with the reasons for activity limitations, this study aimed to investigate the differences in the reasons for activity limitations according to age and sex among community-dwelling stroke survivors. To reflect the stroke survivors’ perspective, the reasons for activity limitations were investigated by asking open-ended questions to survey a wide range of problems, from stroke-related problems to general medical factors. Subjective reporting has the advantage of capturing information regarding the actual difficulties experienced by each individual patient. By understanding these differences, we hope to achieve a more comprehensive understanding of the factors contributing to activity limitations in this population.

## 2. Materials and Methods

### 2.1. Study Design and Participants

In this study, data were obtained from the Korea National Health and Nutrition Examination Survey (KNHANES) operated by the Korea Centres for Disease Control and Prevention (KCDC) [16]. The KNHANES accumulates cross-sectional health-related data through household health interviews, nutritional surveys, and health examinations in specialised mobile examination centres. The KNHANES has been conducted since 1998. We analysed recent data from the KNHANES IV, V, VI, and VII collected from 2007 to 2018. The KCDC has conducted the KNHANES every 3 years from 1998 to 2005 and every year since 2007. A detailed data resource profile of KNHANES data has been previously published [17]. The analyses guide and raw data are publicly accessible on the website in the Korean language (https://knhanes.kdca.go.kr/ accessed on 24 December 2021).

Each survey year, the KNHANES recruits approximately 8000 new participants. The cumulative response rate was 78.4% in the KNHANES IV (2007–2009), 80.8% in the KNHANES V (2010–2012), 78.3% in the KNHANES VI (2013–2015), and 76.6% in the KNHANES VII (2016–2018). The KNHANES was approved by the Institutional Review Board of the KCDC. Written informed consent was obtained from all participants before participation in the KNHANES. The Institutional Review Board of our hospital waived the need for approval because the current study analysed publicly available data. In the present study, individuals who answered ‘yes’ to the question, ‘Have you ever been diagnosed with a stroke by a doctor?’, were defined as stroke survivors [18]. Among the 75,428 (32,778 men and 42,650 women) participants who were aged ≥ 19 years and recruited between 2007 and 2018, 1547 (803 men and 744 women) stroke survivors without missing data were included in the current study.

### 2.2. Variables

Information on the activity limitations experienced by stroke survivors was obtained using a questionnaire [19,20]. The stroke survivors who answered ‘yes’ to the query, ‘Are you currently restricted in your daily life and social activities due to problems with your health, physical disabilities, or mental disabilities?’, were considered stroke survivors with activity limitations [18,19]. If the stroke survivor answered ‘yes’ to the above question, they were instructed to choose the reasons for their activity limitations from the following examples based on their own opinion: stroke, dementia or memory loss, psychiatric problems, visual problems, auditory problems, headache or dizziness, back or neck problems, arthritis, leg pain, arm pain, hypertension, heart disease, diabetes, respiratory problems, gastrointestinal problems, cancer, injuries/trauma, and dental or oral problems. If the reason for activity limitations was not listed in the examples, participants were asked to write it in a free-text field. Since the participants were asked to list all reasons for their activity limitations, multiple responses were possible.

Body mass index (BMI) was calculated from the measured body weight (kg) and height (cm) of participants, who were dressed in light clothing without shoes. Obesity status was categorised according to BMI: underweight (<18.5 kg/m^2^), normal weight (18.5–24.9 kg/m^2^), or obese (≥25 kg/m^2^). The daily alcohol consumption was assessed from a questionnaire that recorded the frequency of drinking and the number of standard drinks consumed per drinking day. Alcohol consumption >10 g/day in women and >20 g/day in men was defined as excessive alcohol consumption. Smoking habits were classified as never before, past, or current. Participants who smoked more than 5 packs of cigarettes (100 cigarettes) in their lifetime and were currently smoking were considered current smokers. Education level (elementary school, middle school, high school, or college/university graduation) and the residence area (urban or rural) were recorded. Participants with systolic blood pressure ≥140 mmHg, diastolic blood pressure ≥90 mmHg, or those taking antihypertensive medication were classified as participants with hypertension. Participants with diabetes were defined as individuals who had a fasting blood glucose level ≥126 mg/dL, were clinically diagnosed by a doctor, were undergoing treatment with oral hypoglycaemic agents, or were undergoing treatment with insulin injections. Participants with cardiovascular diseases were defined as individuals diagnosed with myocardial infarction or angina by a doctor. Participants who have been diagnosed by a doctor with depression, cancer, or arthritis in the past were considered as those with depression, cancer, or arthritis, respectively.

### 2.3. Statistical Analyses

The KNHANES adopts a rolling sampling survey design that applies a complex, stratified, multistage, probability cluster method. Since the KNHANES is a sample survey rather than a complete enumeration survey, expanding the interpretation of the results to the entire Korean population is possible if the analysis is performed by reflecting the complex sampling design. Therefore, the complex sample procedures in SPSS software for Windows, Version 22.0. (IBM Corp., Armonk, NY, USA), were used for all statistical analyses. The categorical values were presented as the weighted percentage (standard error of the percentage (SE)), whereas the continuous variables were presented as the weighted mean ± SE. A value of *p* < 0.05 was considered statistically significant.

The stroke survivors were dichotomised by age into young (<65 years) and older (≥65 years) stroke survivors. To conduct more detailed analyses, we further subdivided the two groups into smaller subgroups with 10-year intervals, except for the youngest and oldest subgroups: younger than 45 years, 45–54 years, 55–64 years, 65–74 years, and 75 years or older. The characteristics of young and older stroke survivors were compared according to sex by using a complex-sample chi-square test. In the present study, the proportion of participants with activity limitations was defined as the weighted percentage of stroke survivors with activity limitations among the total number of stroke survivors. The proportion of stroke survivors with activity limitations and the self-reported reasons for activity limitations were compared according to age groups and sex using the complex-sample chi-square test. As the participants could report multiple reasons for their activity limitations, the total numbers of self-reported reasons for activity limitations were assessed according to age groups and sex using complex-sample general linear models.

## 3. Results

Among stroke survivors aged ≥ 19 years, 51.9% were men, and 65.9% were ≥65 years old. Table 1 shows the characteristics of stroke survivors by age and sex groups (<65 years vs. ≥65 years and men vs. women, respectively). The proportions of excessive alcohol consumption, current smoking status, and higher education level were larger for men than those for women in both young (<65 years) and older (≥65 years) stroke survivors. In addition, the prevalence of individuals with obesity (BMI ≥ 25 kg/m^2^) was higher in female stroke survivors than in male stroke survivors from the older age group. Among young stroke survivors, men exhibited a higher prevalence of diabetes in comparison with women, whereas women exhibited a higher prevalence of depression and arthritis when compared with men. Older female stroke survivors showed significantly higher prevalence of hypertension, depression, and arthritis than male stroke survivors.

### Age and Sex Differences in the Reasons for Self-Reported Activity Limitations

**Figure 1:**
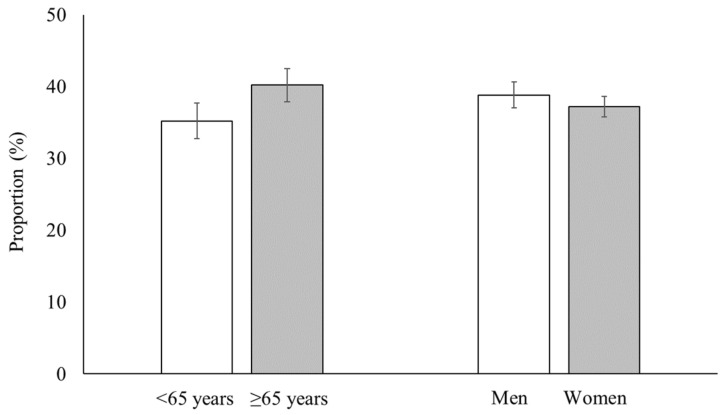
Proportion of people with activity limitations among stroke survivors by age and sex groups.

Among the participating stroke survivors, 38.0% (SE: 1.6%) reported that they had limitations that restricted their daily or social activities. Regarding the proportion of people with activity limitations among stroke survivors, no significant difference was observed between the age or sex groups (Figure 1).

Table 2 and Table 3 show the proportions of each self-reported reason for activity limitations among the total stroke survivors by age groups. When comparing young and older stroke survivors (Table 2), the proportions of dementia or memory loss (1.8% vs. 0.3%; *p* = 0.008), auditory problems (2.0% vs. 0.6%; *p* = 0.024), back or neck problems (9.4% vs. 3.8%; *p* = 0.002), arthritis (7.9% vs. 2.9%; *p* < 0.001), and leg pain (3.5% vs. 0.7%; *p* < 0.001) were significantly larger in older stroke survivors than in younger stroke survivors. The proportions of stroke survivors with activity limitations due to medical problems were not different between young and older stroke survivors.

When comparing the age subgroups (Table 3), significant age-related differences were noted in the prevalence of back or neck problems (*p* = 0.024), arthritis (*p* = 0.002), and leg pain (*p* = 0.013). In addition, individuals under the age of 45 years reported only four reasons for activity limitations, which included stroke (19%), psychiatric problems (4.6%), headaches or dizziness (3.4%), and back or neck problems (4.4%).

As shown in Table 4, in both younger and older age groups, the proportion of individuals with self-reported activity limitations caused by a stroke itself was significantly higher in men than in women (27.1% vs. 15.4% in the younger group, *p* = 0.004; 25.3% vs. 15.5% in the older group; *p* < 0.001). Among younger stroke survivors, women reported more psychiatric problems (5.6% vs. 0.8%; *p* = 0.002), headaches or dizziness (4.5% vs. 0.0%; *p* < 0.001), arthritis (7.3% vs. 0.3%; *p* < 0.001), gastrointestinal problems (2.1% vs. 0.0%; *p* = 0.011), and dental or oral problems (2.4% vs. 0.0%; *p* = 0.021) as reasons for their activity limitations than men. Similarly, among older stroke survivors, women were more likely to report psychiatric problems (2.8% vs. 1.0%; *p* = 0.025), headaches or dizziness (3.1% vs. 1.2%; *p* = 0.032), back or neck problems (12.1% vs. 6.5%; *p* = 0.006), arthritis (13.0% vs. 2.5%; *p* < 0.001), and gastrointestinal problems (1.8% vs. 0.3%; *p* = 0.025) as reasons for their activity limitations, as compared to men.

**Figure 2:**
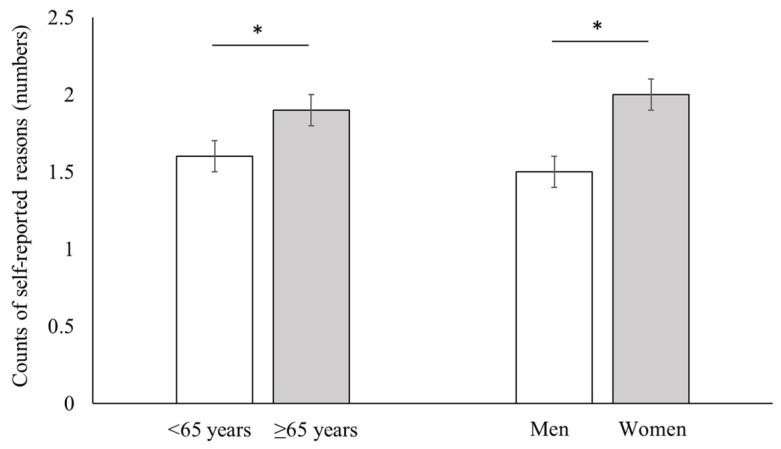
Mean numbers of self-reported reasons for activity limitations in stroke survivors by age and sex groups. * *p* < 0.05.

Figure 2 shows that the weighted mean counts of self-reported reasons for activity limitations were significantly higher in older stroke survivors than in young stroke survivors (1.9 ± 0.1 vs. 1.6 ± 0.1; *p =* 0.010) and in women than in men (2.0 ± 0.1 vs. 1.5 ± 0.1; *p* < 0.001). Females and older stroke survivors tended to report multiple reasons more often for their activity limitations than male and younger stroke survivors, respectively.

## 4. Discussion

Our study identified significant differences in self-reported reasons for activity limitations among community-dwelling stroke survivors stratified by age and sex. Older stroke survivors reported a higher prevalence of dementia or memory loss, auditory problems, back or neck problems, arthritis, and leg pain as reasons for activity limitations than younger stroke survivors. Women reported more activity limitations related to psychiatric problems, headaches or dizziness, back or neck problems, arthritis, gastrointestinal problems, and dental or oral problems than men, while men reported a greater proportion of activity limitations caused by stroke than women. Older and female stroke survivors tended to report multiple reasons for their activity limitations, highlighting the complexity and multifactorial nature of activity limitations in these populations. The findings of the current study will help healthcare providers to administer healthcare that is tailored to the specific needs of stroke survivors with activity limitations while considering their age and sex.

Notable differences were found in the self-reported reasons for activity limitations between older and younger stroke survivors. Specifically, a significantly larger proportion of older stroke survivors reported musculoskeletal problems, auditory problems, and cognitive decline as the reasons for their activity limitations compared with younger survivors. This aligns with previous studies on cause-specific disability in the older adult population, which identified musculoskeletal problems, including back pain and arthritis, as major contributors to disability [21]. Our study also identified a significantly higher prevalence of auditory and memory problems among older stroke survivors than among young survivors, which are consistent with previous reports that hearing impairment is a leading cause of disease burden and an important determinant of the functional capacity of older adults [22,23]. Additionally, although we could not assess the cause of dementia or memory loss in the present study, the high prevalence of cognitive decline and aging-associated memory problems in older stroke survivors can explain the higher prevalence of dementia and memory loss in older stroke survivors than in young stroke survivors [24].

Consistent with findings in previous literature that compared men and women, we found an evident difference in the reasons for activity limitations by sex among stroke survivors [25,26]. Men were more likely to report a previous stroke as the cause of their activity limitations than women. This finding is in agreement with a longitudinal study in Brazil that identified a history of stroke as an important risk factor for disability among men but not among women [25]. Conversely, we found that female stroke survivors were more likely to report several musculoskeletal and medical problems, as well as neurological symptoms, as reasons for their activity limitations than men. These findings are consistent with previous studies that have identified pain and psychiatric problems as significant disabling conditions for women [27,28,29,30]. Women have been found to be over-represented in certain pain conditions, such as headache, abdominal pain, and musculoskeletal pain [27,28]. Furthermore, from a psychological perspective, the proportion of affective disorders, such as anxiety and depression, is approximately double in women compared to in men [30].

The current study revealed that sex differences in activity limitations due to dental or oral problems and back or neck problems were notable when stratified by age. We speculate that the higher prevalence of activity limitations due to dental or oral problems in women than in men may be associated with the tendency of men to ignore oral health more often than women do [31]. A previous study reported no significant differences between men and women in oral health parameters among older adults, which is consistent with the results of our study [32]. Older women reported more back or neck problems as the reasons for activity limitations compared to men. This could be attributed to the role of female sex hormones in causing and exacerbating various musculoskeletal degenerative diseases [33]. Postmenopausal women are particularly susceptible to accelerated disc degeneration, which is why they tend to have low back pain more often than both young and middle-aged women.

The sociodemographic characteristics of the study participants showed significant differences between men and women in terms of educational attainment, excessive alcohol consumption, smoking status, and comorbidities, irrespective of age. These differences may have contributed to the disparities in self-reported reasons for activity limitations between Korean male and female stroke survivors. Sex differences in the contribution of chronic conditions to disability and activity limitations have been consistently reported worldwide [34]. The differences between men and women can also be explained in part by objective health or sex differences from a social perspective [35].

In the current study, the mean number of reasons for activity limitations was higher in the older and female groups than in the younger and male groups, respectively, whereas the proportion of stroke survivors who complained of activity limitations did not differ in terms of age or sex. These results suggest that older or female stroke survivors tend to complain about multiple factors attributable to their activity limitations. The need for comprehensive healthcare for older people with multimorbidity has been continuously raised. The number of chronic conditions associated with ageing was shown to be strongly related to disability [36]. Furthermore, health problems and non-life-threatening disabling conditions, such as musculoskeletal problems and mental disease, were more frequently reported in women [7].

Stroke survivors often experience comorbid lifestyle-related diseases and illnesses, which, if left unaddressed, can exacerbate activity limitations and increase the risk of stroke recurrence. Therefore, attention should be paid to their health-related behaviours. However, since these behaviours are closely related to age and sex, understanding the reasons for activity limitations by subdividing them according to these variables is important [37]. Self-reported reasons for activity limitations reflect an individual’s circumstances and perceptions regarding their real barriers. Our findings are expected to guide the establishment of health-related welfare policies, with an increased focus on addressing difficulties according to age and sex to reduce the activity limitations of stroke survivors and encourage their participation in the community. Tailored care strategies that consider age and sex are essential for decreasing activity limitations in stroke survivors. Our study results indicate that early identification of cognitive impairment and group cognitive therapy are recommended for older adults experiencing early dementia or memory loss and that hearing aids should be provided to those with hearing loss to facilitate communication. Furthermore, considering the high prevalence of musculoskeletal issues in older survivors, classes on proper posture and exercise should be held in the local community and referral systems must be established to facilitate access to necessary treatment. In the case of women, early screening for psychiatric issues is crucial for preventing activity limitations caused by psychiatric problems. Additionally, healthcare professionals should be attentive to the effects of symptoms such as headaches and dizziness on activity limitations and offer appropriate drug therapy to manage their impact on physical activity. Finally, early nutritional screening is necessary to address issues related to gastrointestinal, dental, or oral problems as they may impact nutritional intake, which profoundly influences physical activity [38]. Addressing these concerns can enhance the well-being and activity of community-dwelling stroke survivors while also alleviating the burden on healthcare services.

The results of our study urge age- and sex-specific tailored attention to activity limitations due to musculoskeletal or medical problems as well as neurological sequelae in community-dwelling stroke survivors. As a result of our complex-sampling design, the findings of our study represent the information on the entire Korean community-dwelling stroke survivors. However, this study has several limitations. First, the causal relationship between the variables could not be clarified because of the cross-sectional design. Furthermore, it is difficult to infer the extent of activity limitations caused by stroke because we did not compare the reasons for activity limitations in our study groups with a control group consisting of individuals without a history of stroke. Second, because the date of stroke onset was not available in the database, it was not possible to distinguish between the early and late stages of stroke recovery. Third, the presence of stroke was determined using a questionnaire during the interview and was not objectively confirmed by diagnostic tests such as brain imaging studies. Fourth, data on the objective evaluation of stroke lesions, including ischaemic and haemorrhagic strokes, stroke severity, and post-stroke functions, were not available, and this information was not included in the analyses. Finally, because the reasons for activity limitations were self-reported by stroke survivors and were not based on objective medical judgements, the classification of reasons may be medically ambiguous, some overlap may exist between the reasons, or the interpretations of the results may be limited.

## 5. Conclusions

Although the proportion of activity limitations in stroke survivors did not differ between age and sex groups, the reasons for activity limitations did. Therefore, public health plans for community-dwelling stroke survivors with activity limitations should be tailored based on age and sex, with a focus on older adults and women who tend to have multiple reasons for their activity limitations. Considering that activity limitations are often caused not only by neurological sequelae but also musculoskeletal and medical problems, healthcare professionals should pay attention to various complaints reported by stroke survivors. Further research is needed to investigate the longitudinal effects of these self-reported reasons for activity limitations in stroke survivors.

## Figures and Tables

**Table 1:** Characteristics of community-dwelling stroke survivors aged ≥ 19 years.

Age and Sex Groups	19–64 Years			≥65 Years		
	Men	Women	*p*-Value *	Men	Women	*p*-Value *
Unweighted number (n)	292	235		511	509	
Weighted number (n)	167,743	99,388		169,372	179,989	
Age (years)	54.4 ± 0.6	55.3 ± 0.6	0.269	73.2 ± 0.3	74.0 ± 0.2	0.018
Obesity status (%)			0.624			<0.001
<18.5 kg/m^2^	2.1 (0.8)	1.1 (0.7)		3.9 (1.1)	1.2 (0.4)	
18.5–24.9 kg/m^2^	52.5 (3.3)	55.1 (3.9)		64.4 (2.5)	53.4 (2.7)	
≥25 kg/m^2^	45.3 (3.3)	43.8 (3.9)		31.7 (2.5)	45.4 (2.8)	
Excessive alcohol consumption (%)	15.0 (2.4)	4.5 (1.7)	0.002	7.5 (1.4)	1.0 (0.4)	<0.001
Smoking status (%)			<0.001			<0.001
Current	34.1 (3.3)	5.8 (1.7)		22.2 (2.2)	4.7 (1.1)	
Past	52.9 (3.4)	6.4 (2.0)		62.2 (2.6)	7.8 (1.4)	
Never	13.0 (2.3)	87.8 (2.4)		15.6 (1.8)	87.5 (1.7)	
Education level (%)			<0.001			<0.001
Elementary school	20.2 (2.7)	43.8 (3.9)		49.3 (2.6)	87.4 (1.7)	
Middle school	25.2 (3.2)	21.9 (3.4)		19.1 (2.0)	8.3 (1.5)	
High school	33.8 (3.3)	23.5 (3.5)		22.6 (1.9)	3.8 (1.0)	
College or university	20.8 (2.8)	10.8 (2.4)		9.0 (1.6)	0.5 (0.3)	
Residence area (%)			0.698			0.467
Urban	80.1 (2.7)	78.6 (3.1)		74.8 (2.1)	72.7 (2.3)	
Rural	19.9 (2.7)	21.4 (3.1)		25.2 (2.1)	27.3 (2.3)	
Hypertension (%)	65.2 (3.2)	55.9 (3.8)	0.060	72.5 (2.1)	80.7 (2.1)	0.009
Diabetes (%)	36.0 (3.3)	24.2 (4.0)	0.028	34.4 (2.6)	35.9 (2.7)	0.687
Cardiovascular disease (%)	9.6 (2.0)	8.9 (2.0)	0.801	10.9 (1.5)	10.2 (1.6)	0.772
Depression (%)	3.1 (0.9)	17.3 (3.1)	<0.001	4.0 (0.8)	11.5 (1.5)	<0.001
Cancer (%)	2.3 (1.2)	5.1 (1.9)	0.204	7.4 (1.5)	5.0 (1.1)	0.177
Arthritis (%)	5.2 (1.4)	23.5 (3.3)	<0.001	15.1 (1.9)	49.1 (2.6)	<0.001

Values are presented as the weighted mean ± standard error or weighted percentage (standard error), as appropriate. * *p*-values according to complex-sample chi-square test comparing men and women.

**Table 2:** Self-reported reasons for activity limitations among stroke survivors by age groups.

Age Groups	19–64 Years (n = 527)	≥65 Years (n = 1020)	*p*-Value
Neurological problems			
Stroke	22.7 (2.3)	20.2 (1.4)	0.336
Dementia or memory loss	0.3 (0.2)	1.8 (0.5)	0.008
Psychiatric problems	2.6 (0.8)	1.9 (0.4)	0.433
Visual problems	3.0 (0.9)	4.1 (0.7)	0.385
Auditory problems	0.6 (0.3)	2.0 (0.5)	0.024
Headache or dizziness	1.7 (0.6)	2.2 (0.5)	0.543
Musculoskeletal problems			
Back or neck problems	3.8 (1.1)	9.4 (1.0)	0.002
Arthritis	2.9 (0.7)	7.9 (1.0)	<0.001
Leg pain	0.7 (0.3)	3.5 (0.7)	<0.001
Arm pain	0.6 (0.5)	0.6 (0.2)	0.909
Medical problems			
Hypertension	2.0 (0.5)	2.5 (0.5)	0.471
Heart disease	1.7 (0.6)	1.4 (0.3)	0.725
Diabetes	2.7 (0.7)	2.6 (0.6)	0.948
Respiratory problems	0.8 (0.5)	1.9 (0.4)	0.170
Gastrointestinal problems	0.8 (0.4)	1.1 (0.3)	0.529
Cancer	0.8 (0.6)	0.8 (0.3)	0.906
Injuries/trauma	1.5 (0.7)	2.4 (0.6)	0.330
Dental or oral problems	0.9 (0.5)	2.2 (0.5)	0.131

Values are presented as the weighted percentage (standard error). *p*-values according to the complex-sample chi-square test.

**Table 3:** Self-reported reasons for activity limitations among stroke survivors in age subgroups.

Age Subgroups	<45 Years	45–54 Years	55–64 Years	65–74 Years	≥75 Years	*p*-Value
Neurological problems						
Stroke	19.0 (6.4)	24.1 (4.5)	22.6 (2.7)	20.8 (1.9)	19.6 (2.0)	0.816
Dementia or memory loss	0.0 (0.0)	0.0 (0.0)	0.4 (0.4)	1.1 (0.5)	2.5 (0.8)	0.063
Psychiatric problems	4.6 (3.6)	2.0 (1.1)	2.5 (1.1)	1.9 (0.5)	2.0 (0.6)	0.762
Visual problems	0.0 (0.0)	2.3 (1.8)	3.8 (1.3)	5.5 (1.2)	2.5 (0.8)	0.270
Auditory problems	0.0 (0.0)	0.0 (0.0)	1.0 (0.5)	2.1 (0.7)	1.9 (0.7)	0.276
Headache or dizziness	3.4 (3.3)	2.2 (1.2)	1.1 (0.5)	2.3 (0.7)	2.0 (0.7)	0.721
Musculoskeletal problems						
Back or neck problems	4.4 (3.4)	5.7 (2.8)	2.7 (0.9)	9.5 (1.4)	9.2 (1.4)	0.024
Arthritis	0.0 (0.0)	3.4 (1.4)	3.1 (1.0)	6.3 (1.1)	9.7 (1.6)	0.002
Leg pain	0.0 (0.0)	0.0 (0.0)	1.1 (0.5)	2.6 (0.8)	4.5 (1.0)	0.013
Arm pain	0.0 (0.0)	1.8 (1.7)	0.1 (0.1)	0.9 (0.4)	0.2 (0.2)	0.149
Medical problems						
Hypertension	0.0 (0.0)	1.4 (0.8)	2.6 (0.7)	2.7 (0.7)	2.2 (0.6)	0.537
Heart disease	0.0 (0.0)	1.5 (1.0)	2.0 (0.8)	1.3 (0.4)	1.6 (0.5)	0.774
Diabetes	0.0 (0.0)	1.8 (1.1)	3.5 (1.1)	2.3 (0.8)	2.9 (1.0)	0.609
Respiratory problems	0.0 (0.0)	0.0 (0.0)	1.4 (0.8)	1.5 (0.5)	2.4 (0.7)	0.318
Gastrointestinal problems	0.0 (0.0)	1.3 (0.9)	0.6 (0.4)	0.9 (0.5)	1.2 (0.5)	0.813
Cancer	0.0 (0.0)	0.0 (0.0)	1.3 (1.0)	1.0 (0.5)	0.7 (0.5)	0.730
Injuries/trauma	0.0 (0.0)	2.3 (1.8)	1.3 (0.7)	1.3 (0.6)	3.5 (1.1)	0.328
Dental or oral problems	0.0 (0.0)	0.7 (0.7)	1.2 (0.8)	1.4 (0.5)	3.2 (1.0)	0.176

Values are presented as the weighted percentage (standard error). *p*-values by the complex-sample chi-square test.

**Table 4:** Self-reported reasons for activity limitations among stroke survivors by sex stratified by age.

Age and Sex Groups	19–64 Years			≥65 Years		
	Men	Women	*p*-Value *	Men	Women	*p*-Value *
Neurological problems						
Stroke	27.1 (3.1)	15.4 (2.6)	0.004	25.3 (2.1)	15.5 (1.7)	<0.001
Dementia or memory loss	0.3 (0.3)	0.2 (0.2)	0.579	1.6 (0.6)	2.0 (0.7)	0.600
Psychiatric problems	0.8 (0.5)	5.6 (2.0)	0.002	1.0 (0.4)	2.8 (0.7)	0.025
Visual problems	2.8 (1.1)	3.2 (1.6)	0.825	3.7 (1.1)	4.3 (0.9)	0.682
Auditory problems	0.4 (0.3)	0.9 (0.7)	0.419	2.7 (0.8)	1.4 (0.6)	0.205
Headache or dizziness	0.0 (0.0)	4.5 (1.6)	<0.001	1.2 (0.4)	3.1 (0.9)	0.032
Musculoskeletal problems						
Back or neck problems	2.6 (1.1)	5.9 (2.1)	0.137	6.5 (1.3)	12.1 (1.5)	0.006
Arthritis	0.3 (0.3)	7.3 (1.9)	<0.001	2.5 (0.7)	13.0 (1.7)	<0.001
Leg pain	0.8 (0.5)	0.4 (0.3)	0.371	3.4 (0.9)	3.6 (1.0)	0.901
Arm pain	0.8 (0.8)	0.3 (0.2)	0.336	0.2 (0.1)	0.9 (0.4)	0.056
Medical problems						
Hypertension	1.6 (0.6)	2.6 (0.9)	0.320	1.7 (0.5)	3.2 (0.7)	0.072
Heart disease	1.4 (0.7)	2.1 (0.9)	0.561	1.1 (0.4)	1.8 (0.5)	0.298
Diabetes	3.0 (1.1)	2.2 (0.9)	0.579	2.7 (1.0)	2.5 (0.8)	0.860
Respiratory problems	0.3 (0.3)	1.7 (1.2)	0.133	2.3 (0.7)	1.6 (0.5)	0.467
Gastrointestinal problems	0.0 (0.0)	2.1 (1.0)	0.011	0.3 (0.3)	1.8 (0.6)	0.025
Cancer	1.2 (0.9)	0.0 (0.0)	0.308	1.0 (0.5)	0.7 (0.5)	0.743
Injuries/trauma	1.9 (1.0)	0.8 (0.5)	0.288	2.5 (0.9)	2.3 (0.8)	0.870
Dental or oral problems	0.0 (0.0)	2.4 (1.4)	0.021	2.0 (0.6)	2.4 (0.8)	0.637

Values are presented as the weighted percentage (standard error). * *p*-values according to complex-sample chi-square test comparing men and women.

## Data Availability

The datasets of this study are publicly available (https://knhanes.kdca.go.kr/, accessed on 24 December 2021, in Korean), and the analysed data are available from the corresponding author upon reasonable request.
